# Lead Exposure in Low and Middle-Income Countries: Perspectives and Lessons on Patterns, Injustices, Economics, and Politics

**DOI:** 10.3390/ijerph15112351

**Published:** 2018-10-24

**Authors:** Katarzyna Kordas, Julia Ravenscroft, Ying Cao, Elena V. McLean

**Affiliations:** 1 Department of Epidemiology and Environmental Health, University at Buffalo, Buffalo, NY 14214, USA; julia.ravenscroft@uky.edu; 2 Division of Health Services Policy and Practice, Department of Epidemiology and Environmental Health, University at Buffalo, Buffalo, NY 14214, USA; ycao25@buffalo.edu; 3 Department of Political Science, University at Buffalo, Buffalo, NY 14260, USA; elenamcl@buffalo.edu

**Keywords:** lead, low and middle-income country (LMIC), economic burden, environmental injustice, politics

## Abstract

Lead exposure is a legacy issue that continues to affect vulnerable population groups globally, but particularly in low and middle-income countries (LMICS). We take a multi-disciplinary approach to examine the patterns of lead exposure in these countries, discuss the underlying injustices and socio-political causes, and the economic costs that are associated with exposure. We conclude with some lessons we drew from our discussion of lead across the disciplines and advocate for a number of approaches to solving this ongoing issue. These include (i) biomonitoring that could be integrated into existing health surveys or public health programs targeting young children; (ii) greater civic engagement to push for solutions; and, (iii) environmental control policies that represent a continuum of local, context-specific to broad, national-level, and even global approaches.

## 1. Introduction

Lead exposure among vulnerable population groups, including women of reproductive age and young children, continues to be an important global health problem. Lanphear (2017) names failure to enact environmental standards that would detect lead before children are exposed, eliminate known lead hazards, and ban non-essential uses of lead as some of the reasons for this persistent problem in the United States (U.S.) For these same reasons, and because of growing industrial activities, including the production and use of chemicals [1], high demand for lead-acid batteries [2], and the presence of other vulnerabilities, such as poverty and malnutrition, lead exposure in low and middle-income countries (LMICs) is now more concerning than ever. Many LMIC communities have been grappling with lead contamination for years and are struggling to garner recognition, receive restitutions, or design effective prevention efforts; others are beginning to appreciate its extent and harmful effects. Continued focus on lead occurs in the context of increasing recognition that environmental pollution contributes to the global burden of disease [3].

The issue of lead exposure is not likely to recede from the global arena in the near future, as cycles of legacy exposure intersect with newer exposure pathways such as battery recycling, secondary lead reclamation from existing sources, cottage industries involving lead, as well as ongoing primary production of lead and manufacture of new products. In that sense, lead exposure and poisoning in LMICs is not only an unfinished public health agenda, but also an apparently intractable problem requiring multidisciplinary thinking and creative solutions that go beyond conventional approaches and are tailored to the local context. In this commentary, we offer a multi-disciplinary view on lead exposure in LMICs, grounded in the perspectives we represent: epidemiology/public health, biological anthropology, health economics, and political science. We try to get a handle on patterns of exposure, and consider underlying environmental injustices, associated costs, and political and socio-economic conditions affecting exposure levels. We are particularly interested in framing lead as an issue of health and social inequity. Finally, we draw lessons from the multi-disciplinary, iterative process of writing and talking about lead in LMICs, hoping to spark discussion and renewed commitment to finding effective solutions. 

## 2. Exposure Patterns

Lead is one of the first environmental pollutants to receive widespread attention and it remains the most intensely monitored chemical [4]. In the year 2000, an estimated 120 million people had blood lead levels (BLLs) between 5 and 10 µg/dL, and the majority of them were children [5]. Over 90% of those children lived in LMICs. Although these projections are included in the Global Burden of Disease Project [5], the true extent of lead exposure and poisoning in LMICs is unknown because of non-existent country-level biomonitoring programs. Because target groups or sources of exposure are not well defined, it is very difficult to plan prevention or intervention programs. Lack of biomonitoring data forces reliance on scientific studies, which are seldom representative at the population level. Rather, they select specific regions or groups for study, rely on convenience samples, and are often cross-sectional, thus limiting long-term assessment of changes in BLLs and sources of exposure. Lack of biomonitoring programs presents further challenges. A recent review of studies on children’s environmental health in Latin America revealed that between 1994 and 2014, 70% of the 409 articles originated from Mexico or Brazil, with other countries publishing substantially less [6]. Since 2005, the number of lead-related publications actually declined [6], making scientific studies an unreliable source of information on secular trends and regional patterns. Information published in languages other than English might be missed altogether.

Based on the available scientific evidence, several broad (non-exclusive) categories of affected populations can be identified: (i) those living in areas with diffuse or multiple sources of exposure, particularly urban regions; (ii) those living near toxic sites; and (iii) those engaging in economic activities that put them at high risk of exposure. Additionally, (iv) cultural and subsistence practices may result in lead exposure. Several examples below illustrate the burden of lead exposure among these populations. In many cases, lead is just one of many toxic chemicals in the environment. Furthermore, three issues that are related to lead exposure in LMICs deserve attention. First, a number of studies have documented elevated BLLs among refugees, either prior to or after settling in the U.S. [7,8,9,10,11,12]. These statistics underscore the fact that the global problem of lead can take on very local meaning outside of the countries of origin. Second, lead exposure may be hidden and therefore notoriously difficult to track and prevent through conventional public health programs: it may occur through the use of traditional medicines or employment of rituals practiced by specific cultural groups or select households. Finally, it is worth noting that young children remain the most vulnerable group with respect to lead exposure. Efforts to prevent exposure from daily-use products, like toys [13,14], are needed globally.

### 2.1. Populations with Diffuse or Multiple Sources of Exposure

This exposure pattern has been observed in many LMIC cities undergoing growth and industrialization, including Mexico City, Tehran, Ho Chi Minh City, and Kampala [15,16,17,18]. Cities in China provide a recent example. Although mean BLLs among Chinese children (0–18 years) and the proportion of children with BLLs ≥ 10 μg/dL have declined between 1997 and 2015, notable regional differences persist, with higher concentrations found in areas with heavy industry and looser environmental protection [19]. Despite reductions, contaminated soil, and air pollution from vehicular traffic and widespread industry continue to be strongly correlated with BLLs among children in China [19] and other countries. There is concern over street dust that contains lead and potential exposure via multiple routes, like inhalation and ingestion [16,20]. In some industrial cities, lead concentrations in dust exceed limits set for soil [20]. A related issue, as urban and industrial areas encroach upon or mix with agricultural lands or fisheries, is the contamination of agricultural soils, crops, and aquatic life by lead and other metals [21,22,23,24]. Safeguarding the food supply against contamination by lead and other toxicants will need to be a priority for local governments, planners, and food producers, to protect local as well as global populations, given the increasing globalization of the food industry and trade. 

### 2.2. Populations Living Near Toxic Sites, Including Cities with Little Industrial-Residential Separation

Hazardous waste sites occurring from past or ongoing intensive exploitation of natural resources or poorly controlled industrial and mining activities are an important source of exposure to metals [25,26,27,28,29]. Poor or marginalized populations are more likely to live close to toxic waste sites and suffer the health burden of exposure [30]. The metal smelter in La Oroya, Peru [31] stands as one somber example, but there are many more around the world. Another smelter, the Met-Mex Peñoles in Torreón, Mexico, has been called “one of Mexico’s worst ecological nightmares” [32]. The smelter is one of largest in the world; it also employs a substantial proportion of local residents [32]. When it was established at the end of the 19th century, a wide strip of land separated the smelter from the city, but the space soon filled with homes. In 1970, Mexico’s president formalized the Luis Echeverría settlement, a neighborhood for the working poor, which pushed right against the walls of the complex [32,33,34]. Although the neighborhood was razed around the year 2000, residential homes, several schools, and a soccer field still stand in close proximity to the operation and its slag heap. This is a clear example of how poor urban planning can contribute to grave environmental and health hazards. Since some of the first studies of lead in soil/dust and children’s blood [25,35] in the smelter’s vicinity, lead contamination has decreased [36,37], but remains a problem [38]. 

Hazardous waste sites are numerous in LMICs. In seven Asian countries, 679 sites were screened, mostly (*n* = 498) located in India, Indonesia, and the Philippines [39]. In about one-quarter of these sites, lead was the primary toxicant of concern, but other chemicals were also found. In 82 sites that had soil or water levels above international standards, the predicted BLLs among 0–9 year-old children ranged from 3 to ~60 µg/dL. Another example are the communities of the Corrientes river basin in the Peruvian Amazon, which experienced decades of environmental contamination related to oil exploitation. The overall mean of BLL in these communities was 9.4 µg/dL. Children 0–6 years of age, who comprised 30% of the population, had mean BLL of 7.6 ± 3 µg/dL [40]. Although some communities derive employment opportunities from polluting industries, for others there is no such direct connection. In fact, lead exposure may occur in remote, ostensibly pristine, areas. For example, in the Corrientes river basin, communities that are unaffiliated with the oil industry also had elevated BLLs [40]. These findings underscore the fact that certain groups, either due to poverty, indigenous status, or culturally-based practices experience higher environmental exposures than the general population.

### 2.3. Populations Engaging in “Informal” or “Cottage” Industries That Put Them at High Risk of Exposure

Lead poisoning has been reported in groups that are engaged in activities that produce or use lead. Cottage industry and artisanal production of a variety of wares, as well as jewelry and decorative items, is an important source of lead exposure. For example, women living in Andean communities of Ecuador and engaging in the production and application of lead-based glazes had very high blood and breast milk lead concentrations [41,42]. A survey conducted 10 years later revealed less female involvement in glazing; there was marked decline in exposure, but levels remained elevated [43]. Similar links between the production and application of lead glazes and human exposure were reported in communities of Mexico [44,45] and Brazil [46]. Pantic and colleagues argue that a large proportion of elevated BLLs among Mexican children may be due to use of lead-glazed pottery for cooking and serving food [47].

Many other formal and informal occupational activities are associated with lead exposure in LMICs, including battery manufacture, demolition work, welding, and small businesses repairing automobile radiators [48,49,50,51,52,53]. In a study of children from Cartagena, Colombia, those with highest BLLs lived in a shoreline community with fishing as the primary economic activity; many households made lead sinkers for fishing nets [54]. Similarly, in Chuuk, Federated States of Micronesia, a large proportion of variance in children’s BLLs was explained by parental manufacture of lead fishing weights [14]. Informal or small-scale “industries”, often performed in the home, are also a risk factor for lead exposure and poisoning in Africa. Artisanal ore or gold mining is a particularly important source of exposure. In the Democratic Republic of the Congo, high demand for metals has driven an unregulated small-scale industry in mining, transport and processing of ore [55]. In one case, lead poisoning due to these activities was so severe that it required an emergency response from medical and public health communities. Approximately 400 young children in two villages of Zamfara state, Nigeria, died from what appeared to be unexplained causes. The testing of ~200 children from these communities revealed that 97% had BLLs above 45 µg/dL [56]. Another investigation of Nigeria’s ore mining communities showed that 91% of children who were tested met the criterion for chelation therapy [57]. In both cases, grain grinders were employed to break up gold ore into a fine powder, releasing dust particles and lead into the air and heavily contaminating soil, water, and other surfaces [58]. Thus, human industriousness, combined with lack of knowledge about potential harms, produced disastrous consequences. Water contamination by metals from small-scale mining was also noted in Ghana [59].

Globally, but in particular in Asia and Africa, e-waste recycling, in many cases performed inside home compounds, is an important source of exposure to lead and other chemicals [60,61,62,63,64,65]. In Bangladesh, an estimated 50,000 children are involved in e-waste recycling [66]. Additionally, the burning of paper products, discarded rubber, battery casings, and painted wood for cooking and heating is an exposure pathway in communities with low access to solid fuels or cleaner alternative fuels [67,68]. Clearly, the evaluation of sources of lead exposure in LMICs is complex and likely to differ between areas and population groups, making “one size fits all” prevention programs developed in the U.S. and Western Europe for more universal sources of lead (i.e., paint and gasoline) not fully translatable to the exposure context in LMICs. The very nature of cottage industries makes them difficult to address as sources of lead. In Mexico, for example, between 10 and 50 thousand workshops, which are often based in family homes or compounds, make lead-glazed pottery [69]. Efforts to change their practices are laborious and time-intensive; they happen one workshop at a time. Although interventions have been successful, the sheer size of the “industry”, the fact that the family workshops are scattered across the country, and the existence of important socioeconomic barriers to change, make scaling-up very challenging [70].

### 2.4. Cultural or subsistence practices and lead exposure 

Indigenous peoples, their subsistence practices, and cultural activities deserve special mention. Because of close ties to land and place such practices may place indigenous peoples in greater contact with the outdoor environment and inadvertently at greater risk for exposure to environmental pollutants, like lead [71,72,73,74,75,76,77,78]. For example, rifles and shotguns are used to hunt local game and fowl in many circumpolar cultures and Native peoples are exposed to lead through unintentional ingestion of lead pellets or small pieces of fragmented lead bullets [75,78,79,80,81]. Lead residue from ammunition can potentially contaminate the harvested meat around the entry wound if that part of the kill is not carefully cut away, and hunters can inhale lead dust or fumes during ammunition reloading and detonation [76,77]. Dewailly et al. (2001) found elevated BLLs in Inuit adults from Nunavik in relation to daily consumption of waterfowl and Levesque et al. (2003) were able to link, through isotopic signatures, the lead in cord blood of Nunavik Inuit infants to lead ammunition [74,82]. A multi-community study in northern Quebec (Eeyou Istchee territory) among members of nine Cree Nation communities found that elevated BLLs were associated with being a hunter, the use of firearms with leaded ammunition when hunting, and the consumption of subsistence foods, particularly wild game [75]. In an earlier study of Cree Nation people of the James Bay region of Ontario, the northern Ontario Cree participants had an isotopic ratio signature in their blood similar to that of lead-based ammunition, as compared to a southern Ontario control population with an isotopic signature reflective of atmospheric lead [83]. Resolving public health issues surrounding lead-based ammunition is not straightforward. Hunting and consumption of wild game and birds confers important nutritional benefits, especially in a context where market foods are not widely available, are expensive, and often energy-dense, highly processed foods. Hunting and locally based subsistence activities are an integral part of Native culture and identity. It is critical to incorporate Native perspectives of risk and harm in order to build a consensus regarding approaches to lower exposure to environmental contaminants. such as lead, while preserving cultural well-being and identity and the health benefits that are associated with wild game consumption [84,85].

## 3. Environmental Justice and Lead Exposure in Low and Middle-income Countries

Lead is a significant public health problem in countries with emerging economies [67,86]. In these settings, lead exposure is a consequence of several interacting layers of inequalities, from uneven North-South exchanges and interdependencies in industrial production and trade [3], to country-level economies and politics, to local policies, to social strata and hierarchies, even among the poorest people. Many examples of exposure patterns described above speak to inequalities that are based on socioeconomic status, education, culture, and gender. The consideration of environmental injustices and the unequal exposure of people around the globe to pollutants such as lead is becoming more common [87]. Thus far, much of the work investigating environmental inequity in lead exposure has been conducted in the U.S., where, for instance, the burden of adverse health effects is borne predominantly by low-income residents of older, deteriorating housing stock [88,89,90]. This geospatial clustering is increasingly recognized to represent deep structural inequities rooted in historical processes of residential segregation [87,91,92]. As underlying social, cultural, economic, and historical influences vary across countries, explanatory factors for differential lead exposure within LMICs will likely differ from those in the U.S. Consequently, issues surrounding global environmental injustice will become an area of increasingly active research.

Lead is related to poverty: either because people with low resources live close to point sources of pollution, or, as shown above, they engage in economic activities that expose them to lead, even in their own homes. In the case of work-related exposures, trade-offs are sometimes consciously made—for example, e-waste scavengers in Accra, Ghana may choose this job over less remunerated work in the agricultural sector [93]. Precarious environmental situations that are related to hazardous jobs may be compounded by limited rights, limited physical, social, or health protections, and high level of job insecurity [94]. Poverty may be chronic and even cross-generational [95,96]. The process of lifting families out of poverty is usually gradual, determined by systemic (economic growth, stability, insurance, social protections, etc.), and individual-level factors, and may be interrupted by set-backs [97]. Education, and thereby improved access to information and services, ability to deal with bureaucracies, stronger political voice, and greater resiliency, is one pathway to upward mobility [97], especially for girls. According to the risk-focusing model, however, environmental exposures are “allocated” differentially to communities with certain socially or culturally defined characteristics, such as socioeconomic status; people may enter into or remain in poverty across generations because of previous lead exposure [98]. For people that are exposed to environmental toxicants, where losses of IQ are probable, the road out of poverty through education, resiliency, or formal employment may be difficult. Among adults who had early-life lead exposure, not only lead-related cognitive deficits, but also downward social mobility was noted [99]. Therefore, the societal changes needed for upward mobility ought to be emphasized, particularly because poverty and the hazardous employment choices people make may be brought on by national policies and market forces or international links [93]. 

But, lead should not be regarded exclusively as a “disease of the poor”. Renfew highlights the dangers of relegating lead to the fringes of society [100]. When lead poisoning was “discovered” in the capital of Uruguay, in the working-class neighborhood of La Teja, the community and involved parties (media, politicians, official organs, and agencies of the government) engaged in the process of “othering”—pushing the problem away from themselves, and, eventually, onto the margins of society and the informal settlements where many of the affected individuals lived. The official discourse was quickly distilled into the “Two Ps”: plomo (lead) and pobreza (poverty). As a result, the problem of lead was disconnected from the structural and societal causes—the polluting industries, the economic downturn that precipitated mass migration to Montevideo and occupation of former industrial sites, the lack of employment opportunities—and was attached to the behaviors and personal choices of the victims. What is interesting, the affected community engaged in “othering” as well, creating clear distinctions and hierarchies even within the “poor”, using discriminatory language and laying blame on hygiene, ignorance, and parental neglect. In fact, the majority of the epidemiological research, including our own work [101], has focused on individual or familial predictors of elevated BLLs (maternal age, education, crowding). Some of these are localized indicators of broader societal factors, such as poverty. Nonetheless, they narrowly focus any intervention or prevention measures in terms of behavior change, thus placing the onus on the individual rather than broader institutions or systems. 

In many cases of lead exposure, there is enormous social divide between the people who experience the harm and the individuals and institutions ostensibly meant to protect them. Due to their low political participation or marginalized status, the poor are less likely to be offered help either because they are “invisible” or because their plight does not count as much as strong economic lobbies or other actors in the agendas of politicians. In the case of Met-Mex Peñoles, mothers, concerned for the welfare of their children, protested for months in front of the smelter, the municipality, and even took their case to court. The company initially did little to stop polluting, and later used the Mexican legal system to make the parents’ fight virtually impossible to win [34]. More fundamentally, it may be the health establishment, including ministries and healthcare providers, who act to minimize or avoid dealing with lead [102]. For example, in Uruguay, the Ministries of Health and Economy rejected a decree that would prohibit the production and import of children’s shoes containing lead and cadmium, considering this a “commercial issue… and not a relevant health matter” [103]. In Bangladesh, no laws regulate e-waste import or recycling [66]. Equally, when physicians downplay the importance of lead testing and do not perform screening tests, even upon parents’ request [102], the repercussions are wide-ranging and long-term. 

Some of the examples of exposure patterns cited above also reveal an important gender-based environmental injustice. In Nigeria, ore processing required several steps, and whole communities, including children, participated [56,57,58]. Men processed the ore in central village locations, while women worked in their household compounds, thus bringing the contamination into the home environment. Maternal participation in ore-processing was an important predictor of child BLLs [104] and death. In Bangladesh, women are more likely to participate in e-waste recycling (manual sorting and dismantling) than in electronics repair, which is a male-dominated industry (N. Aich, personal communication). Sadly, women’s participation in informal work that uses or generates toxic chemicals appears to produce greater environmental and health impacts on the household than when men seek that kind of work. 

## 4. Economic and Health Burden of Lead in Low and Middle-income Countries

The large number of people exposed to lead globally from various sources translates into enormous health and economic costs. The consequences of early life lead exposure (starting in utero, through the school years) are wide-ranging and long-lasting, with no lower boundary for the occurrence of harm [105,106]. Environmental contamination also contributes to healthcare expenditures, and LMICs bear a substantial burden of these costs [107]. For example, of the estimated nine-million annual deaths related to pollution (lead, indoor and outdoor air, water, etc.), 15% of all deaths worldwide, more than 90% occur in LMICs [108]. In terms of costs, on the global scale, the total annual, tangible healthcare expenditures attributed to pollution were estimated to range from US$630 billion (upper bound) to US$240 billion (lower bound) or 3–9% of global spending on healthcare in the 2013 reference year [109]. When comparing LMICs with high income countries, although only 14% of global total healthcare spending due to pollution is from LMICs, their relative share of spending for pollution-related illness is a significant part of overall healthcare expenditure [108]. 

The Global Burden of Disease (GBD) study estimates a mean [95% CI] global economic cost of $5 [$3.15–$12.6] billion per year solely for intellectual disability due to lead poisoning [109], and if more comprehensive assessment is used to account for IQ loss during childhood, even LMICs alone would suggest an economic cost of $1.04 [$0.776–$1.237] trillion, nearly 200-fold greater than the GBD approach [109]. Another analysis linked losses in IQ related to lead exposure with losses in economic productivity and lifetime earning potential. In India, for example, lead exposure was estimated to cost $236 billion annually or 5% of GDP, which is equivalent to 121 million lost IQ points [110]. For LMICs altogether, the estimated cost of childhood lead exposure is $977 billion [110]. 

High-income countries have identified and controlled many of the worst problems of environmental pollution, and showed that control is highly cost-effective, especially when one considers the cumulative benefits across generations (e.g., the U.S.’s removal of lead from gasoline in 1976 and the increase in IQ points in the birth cohorts throughout 1990s) [111,112]. But, pollution control has received less local and international attention and resources in LMICs [3]. For example, the total amount that is designated for pollution control is less than 5% of all development spending [111]. In part, due to the urgency of economic development in LMICs, inadequate regulation, informality of many industries, poor surveillance, and improper disposal of contaminants, rapid industrialization in LMICs can result in dangerous exposures and health consequences [113]. Another consideration is that lead tends to affect the poorest of the poor, and, as health effects are often not immediate and occur in contexts with many pressing, competing health concerns (i.e., infectious disease, malnutrition), the problem of lead may be easy for policymakers to ignore. Further discussion of the interplay between resources, regulation, and pollution control in LMICs can be found elsewhere [3,111,112,114]. To understand the apparent inaction in the implementation of prevention and intervention approaches in LMICs, it is critical to understand the political and socio-economic motivators that drive the actors involved in this process, namely industry and policy-makers.

## 5. The Political and Socio-Economic Drivers of Inadequate Pollution Control

Inaction with respect to the problem of lead in LMICs stems from three main challenges. First, there is no single source of lead pollution; in fact, polluters constitute a very large and diverse group of actors. To achieve reductions in lead pollution, these actors would need to overcome free-rider problems. Since action is costly, there is preference to avoid action and minimize associated costs in the hope that others will act and absorb all costs. This attitude results in continued production and the use of lead-based products, and lead exposure remains a health hazard. Polluters, meanwhile, receive benefits from environmental externalities. For example, toxic site operators do not bear the full cost of contamination near toxic sites. 

The second challenge is that, often, the population affected by lead includes women and children, particularly in low-income or marginalized segments of the population [30,41]. These groups are disconnected from each other (for example, some live in remote areas, others are domestic workers), and all tend to be poorly informed about their own exposure, its local prevalence or health consequences. Organizing collective action to reduce lead exposure in such large, dispersed, poorly informed, and poorly mobilized groups is a challenge. Worse, there can be institutional and legal impediments to collective action. For instance, the Mexican legal system does not recognize class-action lawsuits; therefore, parents who sued Met-Mex Peñoles had to submit their lawsuits individually, and prove that the company caused harm to individual children [34]. 

The third category of challenges is linked to patterns of economic development in LMICs and associated sources of lead exposure. According to the environmental risk transition framework, populations in countries with the lowest levels of economic development are at highest risk of household exposures, including activities such as the production of housewares with lead-based glazes or in-home ore processing [115]. As income levels rise, these household hazards decline, but community risks rise because industrial sites and transportation become the dominant sources of exposure. The health impacts of lead exposure reach their highest levels when economic development reaches the interval between 5000 and 9000 $PPP (purchasing power parity) per capita, and decline after that. Finally, when countries reach the high-income group, both household and community hazards decline in importance [115]. These patterns are similar to the general environmental Kuznets curve, which appears to have a turning point at approximately 8000 constant $1990 per capita [116,117,118]. 

Any response to the complex challenge of lead exposure will rely on a combination of political and economic actions. Government policies, such as voluntary cooperation agreements, taxes, and outright bans on leaded gasoline, successfully reduced lead hazards resulting from transportation pollution [118,119]. This suggests that governments can help polluters overcome free-rider problems and force them to absorb a larger share of negative environmental externalities. Another important actor in leading societal response to lead pollution is the civil society and professional community. NGOs in particular can reach broad communities and facilitate learning, information dissemination, public campaigns, etc. to overcome collective action problems among adversely affected groups [120,121]. Social mobilization in defense of the “children of lead” in Torreón, Mexico, illustrates the critical role of civil society: local NGOs joined a local physician and parents in publicizing damaging health effects of unabated pollution from the Peñoles smelter. Their efforts and parent demonstrations put pressure on the Mexican government, which then required Peñoles to reduce pollution [34]. Previous research shows that activities of environmental NGOs (ENGOs) translate into tangible improvements in environmental quality [122]. 

Finally, economic development and reduced income inequality may be essential in increasing societies’ ability to reduce both household and community lead pollution. Even though at lower levels of economic development lead exposure inevitably rises with an increase in the level of economic activity, several countervailing effects help to reverse the trend. First, countries tend to switch to less polluting technologies and economic sectors as their GDP increases [123]. Rising individual incomes strengthen public preference for a cleaner environment and reduce the demand for pollution-intensive goods. Individuals are also in a better position to afford such goods. As a result, trends in economic growth and environmental pollution begin to diverge, resulting in “decoupling” [124]. 

One fundamental political factor enables all three elements of addressing the problem of lead exposure. Civil society action, government responsiveness to pressure from the public, and economic policies spurring economic growth become possible when citizens acquire levers of influence over their governments to hold them accountable for failures to respond. In other words, democratic political institutions enable citizens, NGOs, professional communities, and the private sector to pursue economic development and address the problem of externalities. Economic development appears to benefit significantly from democratic institutions through various channels, including greater human capital, political stability, and economic freedom [125,126]. But, economic development alone is insufficient to reduce exposure to environmental harms: government action is necessary [118]. Advocacy and action driven by professional societies and ENGOs aim to influence government policies, which in turn can address pollution [116]. A UN report indicates that the process of decoupling critically depends on focused government efforts [127]. Such efforts are directly linked to democracy levels [128]. In addition, democracies are more successful in implementing sustainable models of development [129].

The case of La Oroya Antigua, Peru, and its long history of lead exposure from a smelter, illustrates how democratization contributes to environmental improvements. The smelter is nearly 100 years old, but local efforts against pollution only started producing results in the late 1990s, when the government sold the smelter to a private company and required it to curb pollution and implement remediation measures. Civil society, including NGOs, researchers, health professionals and even a Catholic archbishop, mobilized to ensure the company’s compliance with the environmental plan and conducted health studies to show the urgency of clean-up activities [31]. While the company, Doe Run Peru, requested and received an extension for meeting its clean-up targets, it was forced to close the smelter in 2009. The government of Peru attempted to arrange a sale of the smelter, but failed because potential buyers recognized that they would have to comply with air quality regulations and complete the environmental plan for the smelter. The government sought to relax the regulations, but abandoned this plan due to significant opposition. Key developments in Peru’s political and socioeconomic evolution contributed to this outcome. First, with the end of the Fujimori regime, Peru became a full-fledged democracy: the Freedom House considers Peru a “Free” polity, with free and fair elections and robust political competition and participation. Second, the government generally respects freedom of association and does not restrict NGO activities, which facilitates efforts to inform the public regarding pressing social issues. Third, the country has roughly doubled its PPP since 2000, according to the World Bank’s World Development Indicators. Whereas, its PPP stood at $6500 at the end of the Fujimori administration, the 2017 value was over $12,000, which means that Peru has exited the development bracket (5000–9000 $PPP) with the highest level of lead exposure. Finally, the country is successful in its efforts to reduce economic inequality: between 2002 and 2016, the GINI coefficient of income inequality declined from 54 to 44. Reduced inequality can gradually empower groups that suffer from lead exposure the most, enabling them to move away from toxic sites, pursue occupations that do not depend on hazardous industries, and demand better public health outcomes from their representatives. 

## 6. Lessons and Perspectives on Approaches to the Problem of Lead in Low and Middle-income Countries 

Children’s lead exposure is touted as preventable; yet, the experience of recognizing the extent and harms of lead exposure in the U.S. and other high-income countries tells us that it is a lengthy and fraught process. Our iterative process of writing about and discussing lead from several disciplinary perspectives allowed us to draw some lessons that may inform the solutions to the problem. We share these below, fully recognizing that a comprehensive set of recommendations was recently made by an international commission on pollution [3]. In brief, we advocate for (i) biomonitoring programs that on the one hand help pinpoint target groups for intervention and on the other uncover environmental injustices; (ii) greater civic (individuals, groups, ENGOs, etc.) engagement to push for solutions; and, (iii) policies that represent a continuum of local, context-specific to broad, national-level, and global approaches, and that ideally support the equal distribution of outflowing benefits to poor and disenfranchised peoples.

Well-functioning biomonitoring programs are essential for tracking the trends in environmental contamination. Full-scale programs are labor-intensive and costly. The DECOMOPHES pilot collaboration in human biomonitoring among 14 European countries estimated that annual costs of a full-scale program would range €120,000–450,000 per country and €400,000–1,400,000 for central coordination, with additional costs for biobanking. This compares to $5 million/year for the U.S. NHANES and seven million Canadian Dollars/year for the Canadian Health Measures Survey [130]. However, the work of researchers at the National Institute of Public Health in Mexico demonstrates that BLL screening can be implemented into existing nutritional surveys or medical care with the use of portable lead analyzers (M.M. Téllez-Rojo, personal communication). Demographic Health Surveys (DHS), immunization campaigns, home-visiting programs for expectant mothers, or integrated young child feeding or development programs present other large-scale opportunities for the integration of BLL screening. In the absence of any biomonitoring programs, calls to action for individual physicians to provide screening and education to their patients have been issued [131]. While this is commendable, much of the non-urban population in LMICs is not attended by physicians, access to specialized laboratories for sample analysis may be uncertain, and there may be little training within the health system to recognize exposure risks or provide care or education once the results are available.

The healthcare and economic costs of exposure to lead and the benefits of preventing exposure should compel politicians to action. But, it is also evident that politicians tend not to act unless inaction puts their political survival at risk. It is, therefore, essential for the affected populations to recognize the harms of pollution and join forces with NGOs or other civil society actors to put pressure on policy-makers to enact long-term sustainable prevention measures. The final success of the Minamata Convention on Mercury, the international treaty designed to reduce mercury pollution, attests to the power of collaboration across different stake-holders, including the affected communities. Part of the success stems from framing mercury as an issue of environmental injustice with respect to exposures among indigenous peoples. Similarly, civil society involvement can highlight stories of lead poisoning and help to cast it in the light of injustices being done to people who live near sources of pollution or are forced to choose economic activities that increase their exposure. 

However, effective solutions are not solely the remit of private citizens or even organized groups. Both micro and macro approaches are necessary and governmental action, from the highest level to civil servants operating locally, is required. The discussion of socio-economic and political drivers of change in population-wide exposure patterns underscores the need for increased opportunities for education and employment, disposable incomes, mobility, and the participation in governance, particularly among the poorest and most disenfranchised members of society. We noted above that citizens begin demanding cleaner environments when their countries experience economic growth. Because lead exposure disproportionately affects the poor, economic growth needs to be just right—rapid and steady, generating jobs in the sectors that would be reasonably open to people who are poor or have a basic level of education. Then, the poor will benefit by being lifted out of poverty [97], and thus, presumably, having lower risk of harmful exposures. Based on the history of environmental injustice, such policies should particularly protect people, who, based on socioeconomic status, race/ethnicity, indigenous ancestry, or gender, experience the highest levels of pollution and benefit least from economic development.

A persistent impediment to a wide implementation of the precautionary principle, which would guide the protection of vulnerable population from environmental exposures through better regulated industrial activity, has been the perception, based on the “environmental Kuznets hypothesis” that pollution is an unavoidable consequence of economic development. The relationship between pollution and development has come into question [132], as studies demonstrate that mitigation and prevention yield large net gains for both the economy and human health. For example, the removal of lead from gasoline has returned an estimated $200 billion (range, $110 billion–300 billion) to the U.S. economy each year since 1980, an aggregate benefit to date of over $6 trillion through the increased cognitive function and enhanced economic productivity of generations of children [3]. There is also evidence in the U.S. that enacting environmental regulation resulted in the creation of “blue collar” jobs [133]. Thus, the inevitability of pollution during economic development must be further challenged and the case made for greater prosperity and growth. Additionally, the differential meaning of economic growth in the 1950s–1960s, when the focus was on industrial growth and the Kuznets curve was identified, and the current day, when the focus is shifting toward human development, must be recognized. When high-skilled labor becomes a driving force of service-based economies, loss of productivity in the human capital (due to losses in IQ points, for example) may mean losing a narrow competitive advantage. 

When the sources, causes, and patterns of exposure differ between high-income and LMICs, a mixed approach to prevention or mitigation should be considered, wherein the informal sector and culturally-based exposures are emphasized, while traditional public health solutions (phase-out of gasoline, emission controls on industry) are also implemented, as appropriate. In addition to national and international actors, there is a call for local governments to play a role in designing prevention or intervention measures that are specific to the local context [93]. For example, the city of Buffalo in the U.S. recently completed a community needs assessment and issued the “Buffalo Lead Action Plan”, focusing its strategy on local issues—home and rental unit inspections and increased funding for remediation—because living in old housing stock in Buffalo is closely related to elevated BLLs. This is paired with the more traditional, state-, or nation-wide efforts to increase education and BLL screening for children [134]. In LMICs, approaches also need to address exposures in or close to home, but focus on small-scale economic activities or traditional practices, which are targeted appropriately to populations that may have low levels of literacy [135] and limited ability to change the circumstances that drive them to engage in harmful activities. The typical approaches of dissemination through the written word or via health providers are not likely to work in many of these settings. Beyond that, educational campaigns often treat interventions as behavioral and lifestyle choices where, if individuals just had more knowledge, they would make better choices and avoid environmental exposures. This underlying logic overlooks the deep structural disparities that drive people to the margins and engagement with formal or informal sector activities with exposure risks. Educational approaches will do best when integrated with efforts to provide real and accessible alternatives that emphasize systems-level changes that span beyond the public health arena.

## 7. Conclusions

The challenges of addressing lead exposure and poisoning in LMICs result from (i) numerous, diverse, and often small-scale pollution sources (industrial facilities, mines, toxic waste sites, domestic manufacturing workshops, etc.) and environmental externalities benefitting polluters (businesses that emit lead through their regular operations); (ii) collective action problems thwarting action among adversely affected groups; and, (iii) poverty, income inequality, and absence of socially and economically sustainable development. Solutions exist to each of these individual challenges; however, their combination appears to be sufficient to entrench lead exposure as a long-lasting, low-visibility, and low-salience environmental hazard. Therefore, a multi-faceted approach that fits the local context but also addresses global causes of contamination will be needed to address the problem of lead.

## References

[1] OECD OECD Environmnetal Outlook to 2030: Summary in English. http://www.oecd.org/dataoecd/29/33/40200582.pdf.

[2] Economic Development Research Group, Inc (2018). Economic Contribution of the U.S. Lead Battery Industry.

[3] Landrigan P.J., Fuller R., Acosta N.J.R., Adeyi O., Arnold R., Basu N., Bibi Baldé A., Bertollini R., Bose-O’Reilly S., Ivey Boufford J. (2018). The Lancet Commission on pollution and health. Lancet.

[4] Bierkens J., Smolders R., Van Holderbeke M., Cornelis C. (2011). Predicting blood lead levels from current and past environmental data in Europe. Sci. Total Environ..

[5] Prüs-Ustün A., Fewtrell L., Landrigan P.J., Ayuso-Mateos J.L., Ezzati M., Lopez A.D., Rodgers A., Murray C.J.L. (2004). Lead Exposure. Comparative Quantification of Health Risks: Global and Regional Burden of Disease Attributable to Selected Major Risk Factors.

[6] López-Carrillo L., González-González L., Piña-Pozas M., Mérida-Ortega Á., Gamboa-Loira B., Blanco-Muñoz J., Torres-Sánchez L.E., Hurtado-Díaz M., Cortez-Lugo M., Guerra G. (2018). State of children environmental health research in Latin America. Ann. Glob. Health.

[7] Yun K., Matheson J., Payton C., Scott K.C., Stone B.L., Song L., Stauffer W.M., Urban K., Young J., Mamo B. (2016). Health profiles of newly arrived refugee children in the United States, 2006-12. Am. J. Public Health.

[8] Kotey S., Carrico R., Lee Wiemken T., Furmanek S., Bosson R., Nyantakyi F., VanHeiden S., Mattingly W., Zierold K.M. (2018). Elevated blood lead levels by length of time from resettlement to health screening in Kentucky refugee children. Am. J. Public Health.

[9] Eisenberg K.W., van Wijngaarden E., Fisher S.G., Korfmacher K.S., Campbell J.R., Fernandez I.D., Cochran J., Geltman P.L. (2011). Blood lead levels of refugee children resettled in Massachusetts, 2000 to 2007. Am. J. Public Health.

[10] Plotinsky R.N., Straetemans M., Wong L.Y., Brown M.J., Dignam T., Flanders W.D., Tehan M., Azziz-Baumgartner E., Dipentima R., Talbot E.A. (2008). Risk factors for elevated blood lead levels among African refugee children in New Hampshire, 2004. Environ. Res..

[11] Geltman P.L., Brown M.J., Cochran J. (2011). Lead poisoning among refugee children resettled in Massachusetts, 1995 to 1999. Pediatrics.

[12] Mitchell T., Jentes E., Ortega L., Sucosky M.S., Jeffries T., Parr V., Jones W., Brown M.J., Painter J. (2012). Lead poisoning in United States-bound refugee children: Thailand-Burma border, 2009. Pediatrics.

[13] Shen Z., Hou D., Zhang P., Wang Y., Zhang Y., Shi P., O’Connor D. (2018). Lead-based paint in children’s toys sold on China’s major online shopping platforms. Environ. Pollut..

[14] Brown L.M., Kim D., Yomai A., Meyer P.A., Noonan G.P., Huff D., Flanders W.D. (2005). Blood lead levels and risk factors for lead poisoning in children and caregivers in Chuuk State, Micronesia. Int. J. Hyg. Environ. Health.

[15] Havens D., Hong Pham M., Karr C.J., Daniell W.E. (2018). Blood lead levels and risk factors for lead exposure in a pediatric popultion in Ho Chi Minh City, Vietnam. Int. J. Environ. Res. Public Health.

[16] Dehghani S., Moore F., Keshavarzi B., Hale B.A. (2017). Health risk implications of potentially toxic metals in street dust and surface soil in Tehran, Iran. Ecotoxicol. Environ. Safety.

[17] Chuang H.Y., Schwartz J., Gonzales-Cossio T., Lugo M.C., Palazuelos E., Aro A., Hu H., Hernandez-Avila M. (2001). Interrelations of lead levels in bone, venous blood, and umbilical cord blood with exogenous lead exposure through maternal plasma lead in peripartum women. Environ. Health Perspect..

[18] Cusick S., Jaramillo E.G., Moody E.C., Ssemata A.S., Bitwayi D., Lund T.C., Mupere E. (2018). Assessment of blood levels of heavy metals including lead and manganese in healthy children living in the Katanga settlement of Kampala, Uganda. BMC Public Health.

[19] Han Z., Guo X., Zhang B., Liao J., Nie L. (2018). Blood lead levels of children in urban and suburban areas in China (1997–2015): Temporal and spatial variations and influencing factors. Sci. Total Environ..

[20] Tang Z., Chai M., Cheng J., Jin J., Yang Y., Nie Z., Huang Q., Li Y. (2017). Contamination and health risks of heavy metals in street dust from a coal-mining city in eastern China. Ecotoxicol. Environ. Safety.

[21] Liu X., Song Q., Tang Y., Li W., Xu J., Wu J., Wang F., Brookes P.C. (2013). Human health risk assessment of heavy metals in soil-vegetable system: A multi-medium analysis. Sci. Total Environ..

[22] Li Z., Ma Z., van der Kuijp T., Yuan Z., Huang L. (2014). A review of soil heavy metal pollution from mines in China: Pollution and health risk assessment. Sci. Total Environ..

[23] Sharma S., Nagpal A.K., Kaur I. (2018). Heavy metal contamination in soil, food crops and associated health risks for residents of Ropar wetland, Punjab, India and its environs. Food Chem..

[24] Monday Onakpa M., Ayembe Njan A., Chidiebere Kalu O. (2018). A review of heavy metal contamination of food crops in Nigeria. Ann. Glob. Health.

[25] Benin A., Sargent J.D., Dalton M., Roda S. (1999). High concentrations of heavy metals in neighborhoods near ore smelters in northern Mexico. Environ. Health Perspect..

[26] Chen L., Xu Z., Liu M., Huang Y., Fan R., Su Y., Hu G., Peng X., Peng X. (2012). Lead exposure assessment from study near a lead-acid battery factory in China. Sci. Total Environ..

[27] Lubaba Nkulu Banza C., Nawrot T.S., Haufroid V., Decrée S., De Putter T., Smolders E., Ilunga Kabyla B., Numbi Luboya O., Ndala Ilunga A., Mwanza Mutombo A. (2009). High human exposure to cobalt and other metals in Katanga, a mining area of the Democratic Republic of Congo. Environ. Res..

[28] Mees F., Masalehdani M.N.N., De Putter T., D’Hollaner C.D., Van Biezen E., Mujinya B.B., Potdevin J.L., Van Ranst E. (2013). Concentrations and forms of heavy metals around two ore processing sites in Katanga, Democratic Republic of the Congo. J. Afr. Earth Sci..

[29] Grigoryan R., Petrosyan V., Melkonian D.M., Khachadourian V., McCartor A., Crape B. (2016). Risk factors for children’s blood lead levels in metal mining and smelting communities in Armenia: A cross-sectional study. BMC Public Health.

[30] Dowling R., Ericson B., Caravanos J., Grigsby P., Amoyaw-Osei Y. (2015). Spatial associations between contaminated land and sociodemographics in Ghana. Int. J. Environ. Res. Public Health.

[31] Fraser B. (2009). La Oroya’s legacy of lead. Environ. Sci. Technol..

[32] Smith J.F. (1999). A Mexican City awakes to an ecological nightmare. Los Angeles Times.

[33] Preston J. (1999). Lead dust in the wind withers Mexican children. The New York Times.

[34] Díez J., Rodríguez R., Carruthers D.V. (2008). Environmental justice in Mexico: The Peñoles case. Environmental Justice in Latin America: Problems, Promise, and Practice.

[35] Garcia Vargas G.G., Rubio Andrade M., Del Razo L.M., Borja Aburto V., Vera Aguilar E., Cebrian M.E. (2001). Lead exposure in children living in a smelter community in Region Lagunera, Mexico. J. Toxicol. Environ. Health.

[36] Rubio-Andrade M., Valdés-Pérezgasga F., Alonso J., Rosado J.L., Cebrián M.E., García-Vargas G.G. (2011). Follow-up study on lead exposure in children living in a smelter community in northern Mexico. Environ. Health.

[37] Garcia-Vargas G.G., Rothenberg S.J., Silbergeld E.K., Weaver V., Zamoiski R., Resnick C., Rubio-Andrade M., Parsons P.J., Steuerwald A.J., Navas-Acién A. (2014). Spatial clustering of toxic trace elements in adolescents around the Torreón, Mexico lead-zinc smelter. J. Expo. Sci. Environ. Epidemiol..

[38] Soto-Jiménez M.F., Flegal A.R. (2011). Childhood lead poisoning from the smelter in Torreón, México. Environ. Res..

[39] Caravanos J., Chatham-Stephens K., Bret E., Landrigan P.J., Fuller R. (2013). The burden of disease from pediatric exposure at hazardous waste sites in 7 Asian countries. Environ. Res..

[40] Anticona C., Berghdal I.A., Lundh T., Alegre Y., San Sebastian M. (2011). Lead exposure in indigenous communities of the Amazon basin, Peru. Int. J. Hyg. Environ. Health.

[41] Butler Walker J., Houseman J., Seddon L., McMullen E., Tofflemire K., Mills C., Corriveau A., Weber J.P., LeBlanc A., Walker M. (2006). Maternal and umbilical cord blood levels of mercury, lead, cadmium, and essential trace elements in Arctic Canada. Environ. Res..

[42] Iqbal S., Blumenthal W., Kennedy C., Yip F.Y., Pickard S., Flanders W.D., Loringer K., Kruger K., Caldwell K.L., Jean Brown M. (2009). Hunting with lead: Association between blood lead levels and wild game consumption. Environ. Res..

[43] Johansen P., Pedersen H.S., Asmund G., Riget F. (2006). Lead shot from hunting as a source of lead in human blood. Environ. Pollut..

[44] Tsuji L.J., Wainman B.C., Martin I.D., Weber J.P., Sutherland C., Liberda E.N., Nieboer E. (2008). Elevated blood-lead levels in first nation people of Northern Ontario Canada: Policy implications. Bull. Environ. Contam. Toxicol..

[45] Dewailly E., Ayotte P., Bruneau S., Lebel G., Levallois P., Weber J.P. (2001). Exposure of the Inuit population of Nunavik (Arctic Quebec) to lead and mercury. Arch. Environ. Health.

[46] Liberda E.N., Tsuji L.J.S., Martin I.D., Ayotte P., Robinson E., Dewailly E., Nieboer E. (2018). Source identification of human exposure to lead in nine Cree Nations from Quebec, Canada (Eeyou Istchee territory). Environ. Res..

[47] Verbrugge L.A., Wenzel S.G., Berner J.E., Matz A.G. (2009). Human exposure to lead from ammunition in the circumpolar north. Ingestion of Lead from Spent Ammunition: Implications for Wildlife and Humans.

[48] Tsuji L.J.S., Nieboer E., Karagatzides J.D., Hanning R.M., Katapatuk B. (1999). Lead shot contamination in edible portions of game birds and its dietary implications. Ecosyst. Health.

[49] Dewailly E., Levesque B., Duchesene J.F., Dumas P., Scheuhammer A., Gariepy C., Rhainds M., Proulx J.F. (2000). Lead shot as a source of lead poisoning in the Canadian Arctic. Epidemiology.

[50] Hunt W.G., Watson R.T., Oaks J.L., Parish C.N., Burnham K.K., Tucker R.L., Belthoff J.R., Hart G. (2009). Lead bullet fragments in venison from rifle-killed deer: Potential for human dietary exposure. PLoS ONE.

[51] Tsuji L.J., Wainman B.C., Martin I.D., Sutherland C., Weber J.P., Dumas P., Nieboer E. (2008). Lead shot contribution to blood lead of First Nations people: The use of lead isotopes to identify the source of exposure. Sci. Total Environ..

[52] Couture A., Levesque B., Dewailly E., Muckle G., Dery S., Proulx J.F. (2012). Lead exposure in Nunavik: From research to action. Int. J. Circumpolar Health.

[53] Levesque B., Duchesne J.F., Gariepy C., Rhainds M., Dumas P., Scheuhammer A.M., Proulx J.F., Dery S., Muckle G., Dallaire F. (2003). Monitoring of umbilical cord blood lead levels and sources assessment among the Inuit. Occup. Environ. Med..

[54] Tsuji L.J., Wainman B.C., Martin I.D., Sutherland C., Weber J.P., Dumas P., Nieboer E. (2008). The identification of lead ammunition as a source of lead exposure in First Nations: The use of lead isotope ratios. Sci. Total Environ..

[55] Arquette M., Cole M., Cook K., LaFrance B., Peters M., Ransom J., Sargent E., Smoke V., Stairs A. (2002). Holistic risk-based environmental decision making: A Native perspective. Environ. Health Perspect..

[56] King U., Furgal C. (2014). Is hunting still healthy? Understanding the interrelationships between indigenous participation in land-based practices and human-environmental health. Int. J. Environ. Res. Public Health.

[57] Counter S.A., Buchanan L.H., Ortega F. (2004). Current pediatric and maternal lead levels in blood and breast milk in Andean inhabitants of a lead-glazing enclave. J. Occup. Environ. Med..

[58] Counter S.A., Buchanan L.H., Ortega F. (2007). Lead concentrations in maternal blood and breast milk and pediatric blood of Andean villagers: 2006 follow-up investigation. J. Occup. Environ. Med..

[59] Counter S.A., Buchanan L.H., Ortega F., Chriboga R., Correa R., Collaguaso M.A. (2014). Lead levels in the breast milk of nursing Andean mothers living in a lead-contaminated environment. J. Toxicol. Environ. Health.

[60] Hernandez-Avila M., Romieu I., Rios C., Rivero A., Palazuelos E. (1991). Lead-glazed ceramics as major determinants of blood lead levels in Mexican women. Environ. Health Perspect..

[61] Farías P., Álamo-Hernández U., Mancilla-Sánchez L., Texcalac-Sangrador J.L., Carrizales-Yáñez L., Riojas-Rodríguez H. (2014). Lead in school children from Morelos, Mexico: Levels, sources and feasible interventions. Int. J. Environ. Res. Public Health.

[62] Costa R.G., Araújo C.F.D.S., Ferreol Bah A.H., Junior E.A.G., Rodrigues Y.J.M., Menezes-Filho J.A. (2018). Lead in mangrove root crab (Goniopsis cruentata) and risk assessment due to exposure for estuarine villagers. Food Addit. Contam..

[63] Pantic I., Tamayo-Ortiz M., Rosa-Parra A., Bautista-Arredondo L., Wright R.O., Peterson K., Schnaas L., Rothenberg S.J., Hu H., Téllez-Rojo M.M. (2018). Children’s blood lead concentrations from 1988 to 2015 in Mexico City: The contribution of lead in air and traditional lead-glazed ceramics. Int. J. Environ. Res. Public Health.

[64] ISEE (2015). Commentary: ISEE Call for Action for Global Control of Lead Exposure to Eliminate Lead Poisoning. Epidemiology.

[65] Paoliello M.M., De Capitani E.M. (2005). Environmental contamination and human exposure to lead in Brazil. Rev. Environ. Contam. Toxicol..

[66] Brown V.J. (2004). Electronics, lead, and landfills. Environ. Health Perspect..

[67] Hellstrom-Lindberg E., Bjorklund A., Karlson-Stiber C., Harper P., Selden A.I. (2006). Lead poisoning from souvenir earthenware. Int. Arch. Occup. Environ. Health.

[68] Mathee A., von Schirnding Y., Montgomery M., Rollin H. (2004). Lead poisoning in South African children: The hazard is at home. Rev. Environ. Health.

[69] Li G.J., Zhang L.L., Lu L., Wu P., Zheng W. (2004). Occupational exposure to welding fume among welders: Alterations of manganese, iron, zinc, copper, and lead in body fluids and the oxidative stress status. J. Occup. Environ. Med..

[70] Olivero-Verbel J., Duarte D., Echenique M., Guette J., Johnson-Restrepo B., Parsons P.J. (2007). Blood lead levels in children aged 5-9 years living in Cartagena, Colombia. Sci. Total. Environ..

[71] Nemery B., Banza Lubaba Nkulu C. (2018). Assessing exposure to metals using biomonitoring: Achievements and challenges experienced through surveys in low- and middle-income countries. Toxicol. Lett..

[72] Dooyema C.A., Neri A., Lo Y.C., Durant J., Dargan P.I., Swarthout T., Biya O., Gidado S.O., Haladu S., Sani-Gwarzo N. (2012). Outbreak of fatal childhood lead poisoning related to artesanal gold mining in Northwestern Nigeria, 2010. Environ. Health Perspect..

[73] Olamide Ajumobi O., Tosofo A., Yango M., Kamweli Aworh M., Nkiruka Anagbogu I., Mohammed A., Umar-Tsafe N., Mohammed S., Abdullahi M., Davis L. (2014). High concentration of blood lead levels among young children in Bagega community, Zamfara-Nigeria and the potential risk factor. Pan Afr. Med. J..

[74] Kaufman J.A., Brown C.J., Umar-Tsafe N.T., Bashir Abdullahi M., Getso K.I., Kaita I.M., Bako Sule B., Ba’aba A., Davis L., Nguku P.M. (2016). Prevalence and risk factors of elevated blood lead in children in gold ore processing communities, Zamfara, Nigeria, 2012. J. Health Pollut..

[75] Cobbina S.J., Duwiejuah A.B., Quansah R., Obiri S., Bakobie N. (2015). Comparative assessment of heavy metals in drinking water sources in two small-scale mining communities in Northern Ghana. Int. J. Environ. Res. Public Health.

[76] Amankwaa E.F., Adovor Tsikudo K.A., Bowman J.A. (2017). ‘Away’ is a place: The impact of electronic waste recycling on blood lead levels in Ghana. Sci. Total Environ..

[77] Srigboh R.K., Basu N., Stephens J., Asampong E., Perkins M., Neitzel R.L., Fobil J. (2016). Multiple elemental exposures amongst workers at the Agbogbloshie electronic waste (e-waste) site in Ghana. Chemosphere.

[78] Pascale A., Sosa A., Bares C., Battocletti A., Moll M.J., Pose D., Laborde A., González H., Feola G. (2016). E-Waste Informal Recycling: An Emerging Source of Lead Exposure in South America. Ann. Glob. Health.

[79] Kyere V.N., Grevem K., Atiemo S.M., Ephraim J. (2017). Spatial assessment of potential ecological risk of heavy metals in soils from informal e-waste recycling in Ghana. Environ. Health Toxicol..

[80] Chen A., Dietrich K.N., Huo X., Ho S. (2011). Developmental neurotoxicants in e-waste: An emerging health concern. Environ. Health Perspect..

[81] Kumar Awasthi A., Zeng Z., Li J. (2016). Environmental pollution of electronic waste recycling in India: A critical review. Environ. Pollut..

[82] ESDO (Environment and Social Development Organization) (2014). Magnitude of the Flow of e-Waste in Bangladesh.

[83] Tong S., von Schirnding Y.E., Prapamontol T. (2000). Environmental lead exposure: A public health problem of global dimensions. Bull. World Health Organ..

[84] Nriagu J.O., Blankson M.L., Ocran K. (1996). Childhood lead poisoning in Africa: A growing public health problem. Sci. Total Environ..

[85] Earth. P. Fact Sheet: Mexico’s 500-Year-Old Problem (Infographic). http://www.pureearth.org/blog/fact-sheet-mexicos-500-year-old-problem-infographic/.

[86] Tamayo-Ortiz M., Navia-Antezana J. (2018). Reduced lead exposure following a sensitization program in rural family homes producing traditional Mexican ceramics. Ann. Glob. Health.

[87] Meyer P.A., Brown M.J., Falk H. (2008). Global approach to reducing lead exposure and poisoning. Mutat. Res..

[88] Leech T.G., Adams E.A., Weathers T.D., Staten L.K., Filippelli G.M. (2016). Inequitable Chronic Lead Exposure: A Dual Legacy of Social and Environmental Injustice. Fam. Commun. Health.

[89] Jacobs D.E. (2011). Environmental health disparities in housing. Am. J. Public Health.

[90] Bernard S.M., McGeehin M.A. (2003). Prevalence of blood lead levels >or= 5 micro g/dL among US children 1 to 5 years of age and socioeconomic and demographic factors associated with blood of lead levels 5 to 10 micro g/dL, Third National Health and Nutrition Examination Survey, 1988–1994. Pediatrics.

[91] Sadler R.C., LaChance J., Hanna-Attisha M. (2017). Social and Built Environmental Correlates of Predicted Blood Lead Levels in the Flint Water Crisis. Am. J. Public Health.

[92] Gee G.C., Payne-Sturges D.C. (2004). Environmental health disparities: A framework integrating psychosocial and environmental concepts. Environ. Health Perspect..

[93] Moody H., Grady S.C. (2017). Lead emissions and population vulnerability in the Detroit (Michigan, USA) Metropolitan Area, 2006-13: A spatial and temporal analysis. Int. J. Environ. Res. Public Health.

[94] Oteng-Ababio M. (2012). When necessity begets ingenuity: E-waste scavenging as a livelihood strategy in Accra, Ghana. Afr. Stud. Quart..

[95] Muntaner C., Solar O., Vanroelen C., Martínez J.M., Vergara M., Santana V., Castedo A., Kim I.H., Benach J., Network E. (2010). Unemployment, informal work, precarious employment, child labor, slavery, and health inequalities: Pathways and mechanisms. Int. J. Health Serv..

[96] Bird K. (2007). The Intergenerational Transmission of Poverty: An Overview.

[97] Corak M. (2013). Income inequality, equality of opportunity, and intergenerational mobility. J. Econ. Perspect..

[98] Miller R., Methenge M., Bird K., Karin F.Z., Gitau R., Kaloi Nteza E. (2011). Ascending out of Poverty: An Analysis of Family Histories in Kenya.

[99] Schell L. (1997). Culture as a stressor: A revised model of biocultural interaction. Am. J. Phys. Anthro..

[100] Reuben A., Caspi A., Belsky D.W., Broadbent J., Harrington H., Sugden K., Houts R.M., Ramrakha S., Poulton R., Moffitt T.E. (2017). Association of childhood blood lead levels with cognitive function and socioeconomic status at age 38 years and with IQ change and socioeconomic mobility between childhood and adulthood. JAMA.

[101] Renfrew D. (2013). “We are not marginals”. The cultural politics of lead poisoning in Montevideo, Uruguay. Latin Am. Perspect..

[102] Queirolo E.I., Ettinger A.S., Stoltzfus R.J., Kordas K. (2010). Association of anemia, child and family characteristics with elevated blood lead concentrations in preschool children from Montevideo, Uruguay. Arch. Environ. Occup. Health.

[103] Renfrew D. (2017). Spectral science: Tracing the conflict zones of Uruguayan lead poisoning. Cult. Theory Crit..

[104] Taylor M.P., Winder C., Lanphear B.P. (2014). Australia’s leading public health body delays action on the revision of the public health goal for blood lead exposures. Environ. Int..

[105] (2015). El MSP rechaza decreto que prohíbe el plomo en el calzado. El País.

[106] Bashir M., Umar-Tsafe N., Getso K., Kaita I.M., Nasidi A., Sani-Gwarzo N., Nguku P., Davis L., Brown M.J. (2014). Assessment of blood lead levels among children aged ≤ 5 years—Zamfara State, Nigeria, June-July 2012. MMWR.

[107] Jusko T.A., Henderson C.R., Lanphear B.P., Cory-Slechta D.A., Parsons P.J., Canfield R.L. (2008). Blood lead concentrations <10 microg/dL and child intelligence at 6 years of age. Environ. Health Perspect..

[108] Lanphear B.P., Hornung R., Khoury J., Yolton K., Baghurst P., Bellinger D.C., Canfield R.L., Dietrich K.N., Bornschein R., Greene T. (2005). Low-level environmental lead exposure and children’s intellectual function: An international pooled analysis. Environ. Health Perspect..

[109] Landrigan P.J., Fuller R., Hu H., Caravanos J., Cropper M.L., Hanrahan D., Sandilya K., Chiles T.C., Kumar P., Suk W.A. (2018). Pollution and global health—An agenda for prevention. Environ. Health Perspect..

[110] Preker A.S., Adeyi O.O., Lapetra M.G., Simon D.C., Keuffel E. (2016). Health care expenditures associated with pollution: Exploratory methods and findings. Ann. Glob. Health.

[111] Grandjean P., Bellanger M. (2017). Calculation of the disease burden associated with environmental chemical exposures: Application of toxicological information in health economic estimation. Environ. Health.

[112] Attina T.M., Trasande L. (2013). Economic costs of childhood lead exposure in low- and middle-income countries. Environ. Health Perspect..

[113] Landrigan P.J., Fuller R. (2016). Pollution, health and development: The need for a new paradigm. Rev. Environ. Health.

[114] Trasande L., Massey R.I., DiGangi J., Geiser K., Olanipekun A.I., Gallagher L. (2011). How developing nations can protect children from hazardous chemical exposures while sustaining economic growth. Health Aff..

[115] Caravanos J., Carrelli J., Dowling R., Pavilonis B., Ericson B., Fuller R. (2016). Burden of disease resulting from lead exposure at toxic sites in Argentina, Mexico and Uruguay. Environ. Health.

[116] Landrigan P.J., Fuller R. (2012). Environmental pollution: An enormous and invisible burden on health systems in low-and middle-income countries. World Hosp. Health Serv..

[117] Smith K.R., Ezzati M. (2005). How environmental health risks change with development: The epidemiologic and environmental risk transitions revisited. Ann. Rev. Environ. Res..

[118] Grossman G.M., Krueger A.B. (1996). The inverted-U: What does it mean?. Environ. Dev. Econ..

[119] Harbaugh W.T., Levinson A., Wilson D.M. (2002). Reexamining the empirical evidence for an environmental Kuznets curve. Rev. Econ. Stat..

[120] Hilton F.H., Levinson A. (1998). Factoring the environmental Kuznets curve: Evidence from automotive lead emissions. J. Environ. Econ. Manag..

[121] Wurzel R., Jordan A., Zito A., Brückner L. (2003). From high regulatory state to social and ecological market economy? New environmental policy instruments in Germany. Environ. Polit..

[122] Selden T.M., Forrest A.S., Lockhart J.E. (1999). Analyzing the reductions in U.S. air pollution emissions: 1970 to 1990. Land Econ..

[123] Finger M., Princen T. (2013). Environmnetal NGOs in World Politics: Linking the Local and the Global.

[124] Binder S., Neumayer E. (2005). Environmental pressure group strength and air pollution: An empirical analysis. Ecol. Econ..

[125] Cole M.A. (2000). Air pollution and ‘ditty industries’: How and why does the composition of manufacturing output change with economic development?. Environ. Resour. Econ..

[126] Harris J.M., Roach B. (2013). Environmental and Natural RESOURCE Economics: A Contemporary Approach.

[127] Doucouliagos H., Ulubaşoğlu M.A. (2008). Democracy and economic growth: A meta-analysis. Am. J. Polit. Sci..

[128] Acemoglu D., Naidu S., Restrepo P., Robinson J.A. (2014). Democracy Does Cause Growth.

[129] Fischer-Kowalski M., Swilling M. (2011). Decoupling: Natural Resource Use and Environmental Impacts from Economic Growth.

[130] Lijphart A. (1999). Patterns of Democracy: Government Forms and Performance in Thirty-Six Countries.

[131] Ward H. (2008). Liberal democracy and sustainability. Environ. Polit..

[132] Joas A., Knudsen L.E., Kolossa-Gehring M., Sepai O., Casteleyn L., Schoeters G., Angerer J., Castaño A., Aerts D., Biot P. (2015). Policy recommendations and cost implications for a more sustainable framework for European human biomonitoring surveys. Environ. Res..

[133] Feiock R.C., Stream C. (2001). Environmental protection versus economic development: A false trade-off?. Public Admin. Rev..

[134] Goodstein E. (1995). Jobs or the environment? No trade-off. Challenge.

[135] Gardner K. (2018). Renewing Our Pledge: A Path to Ending Lead Poisoning of Buffalo’s Most Vulnerable Citizens.

